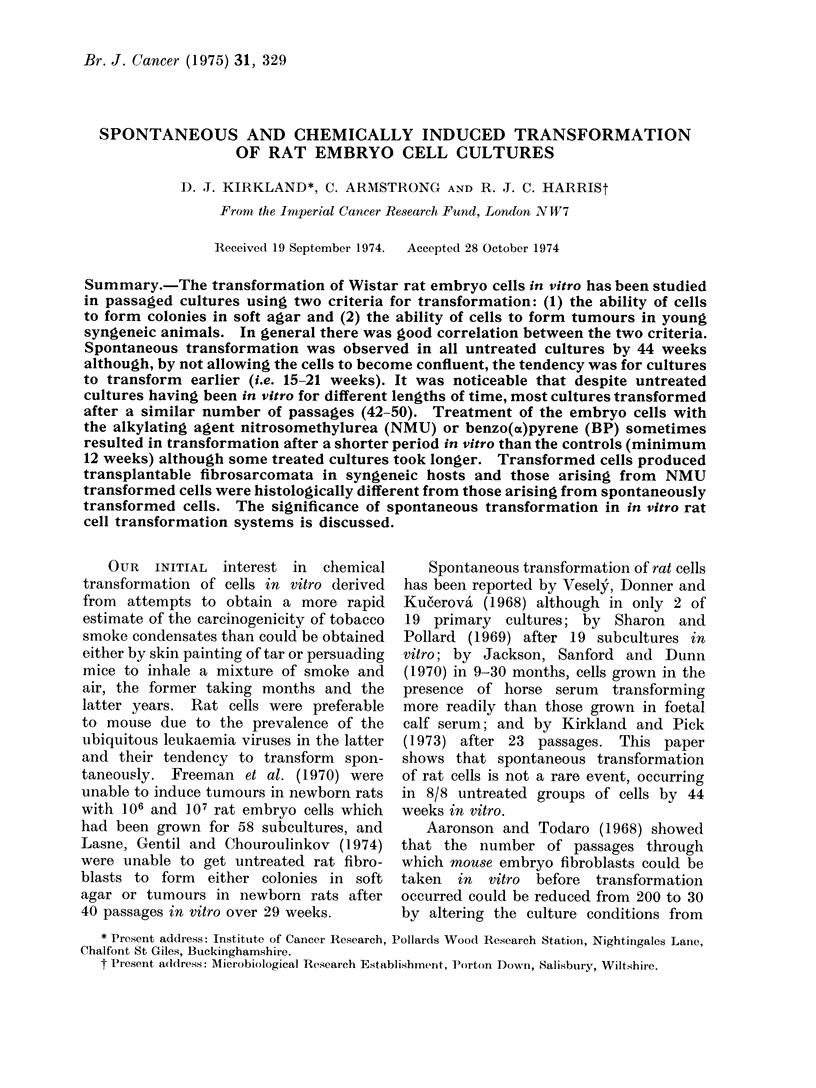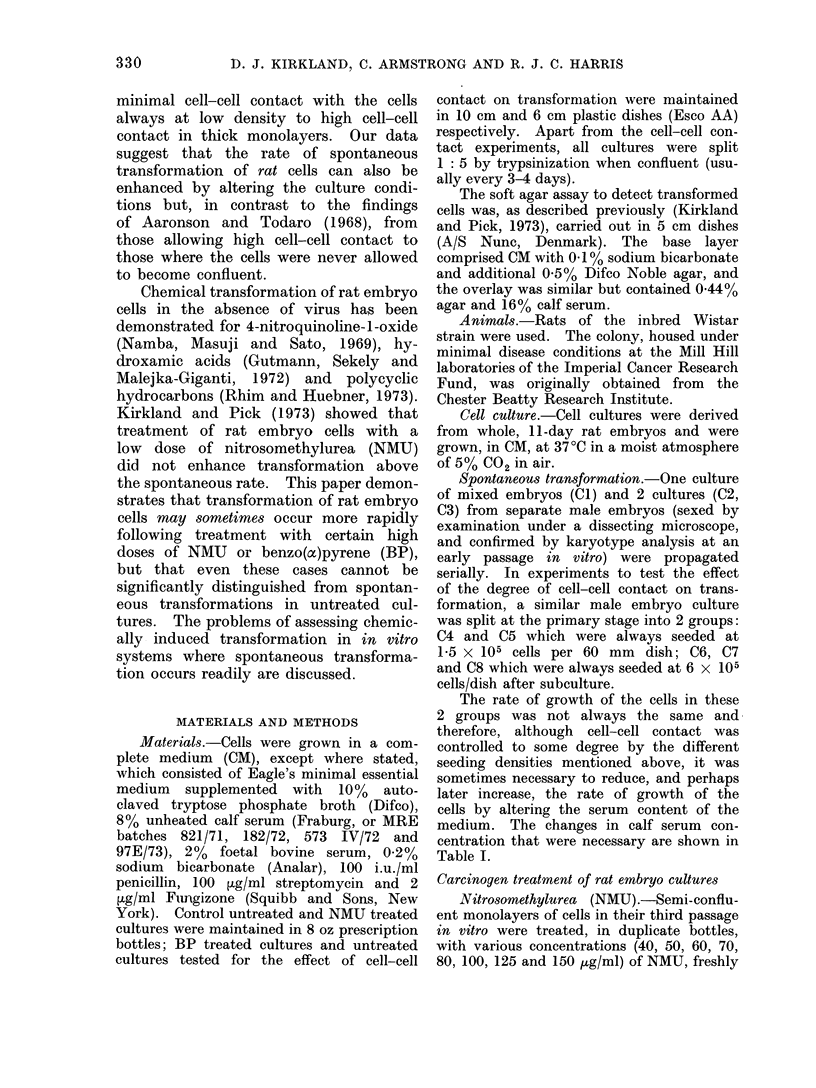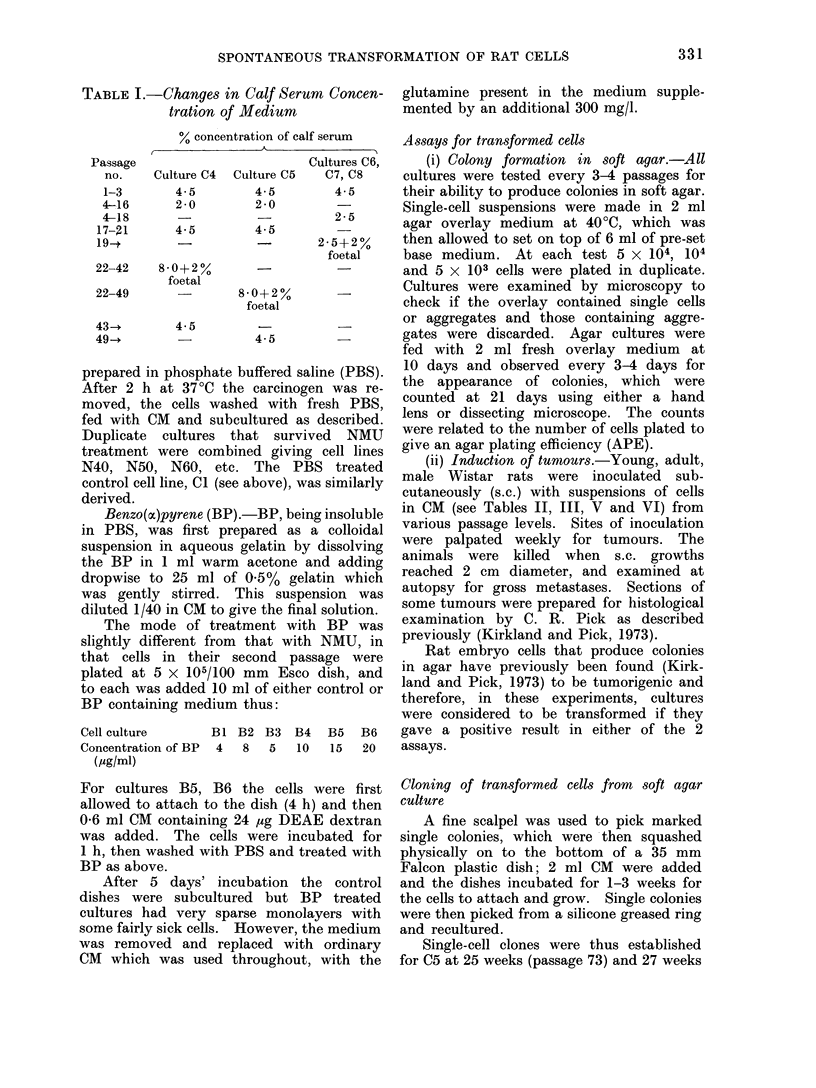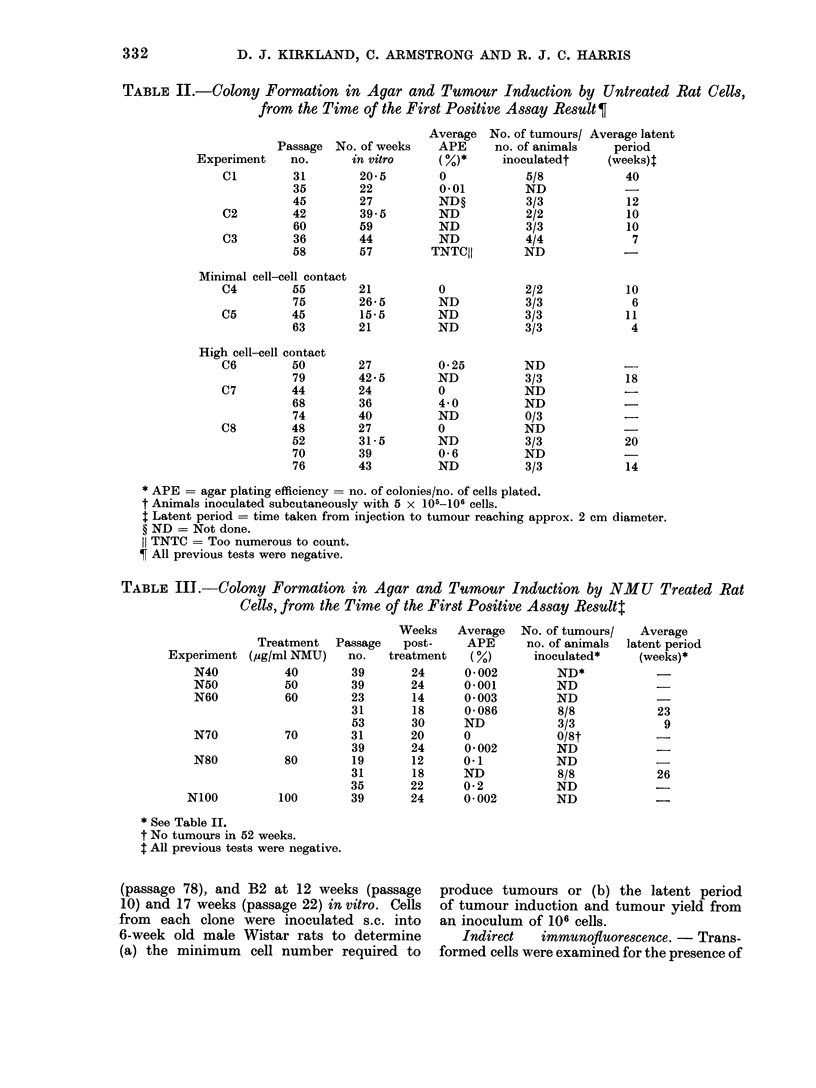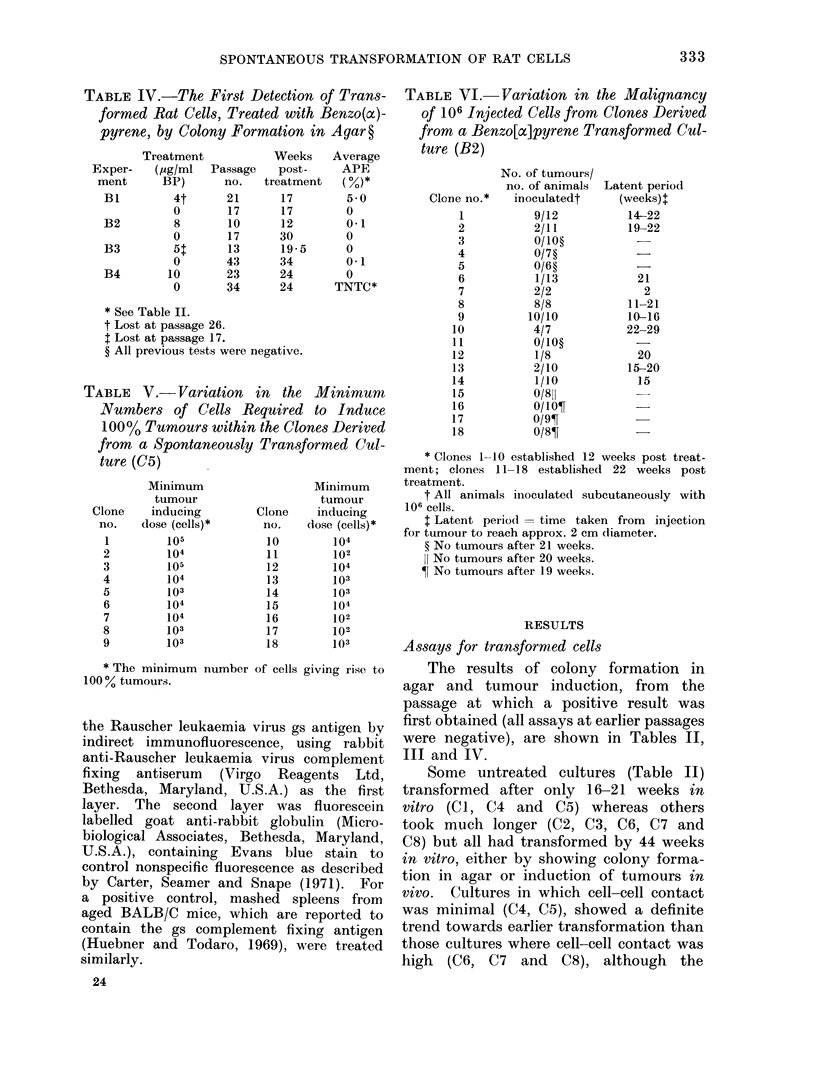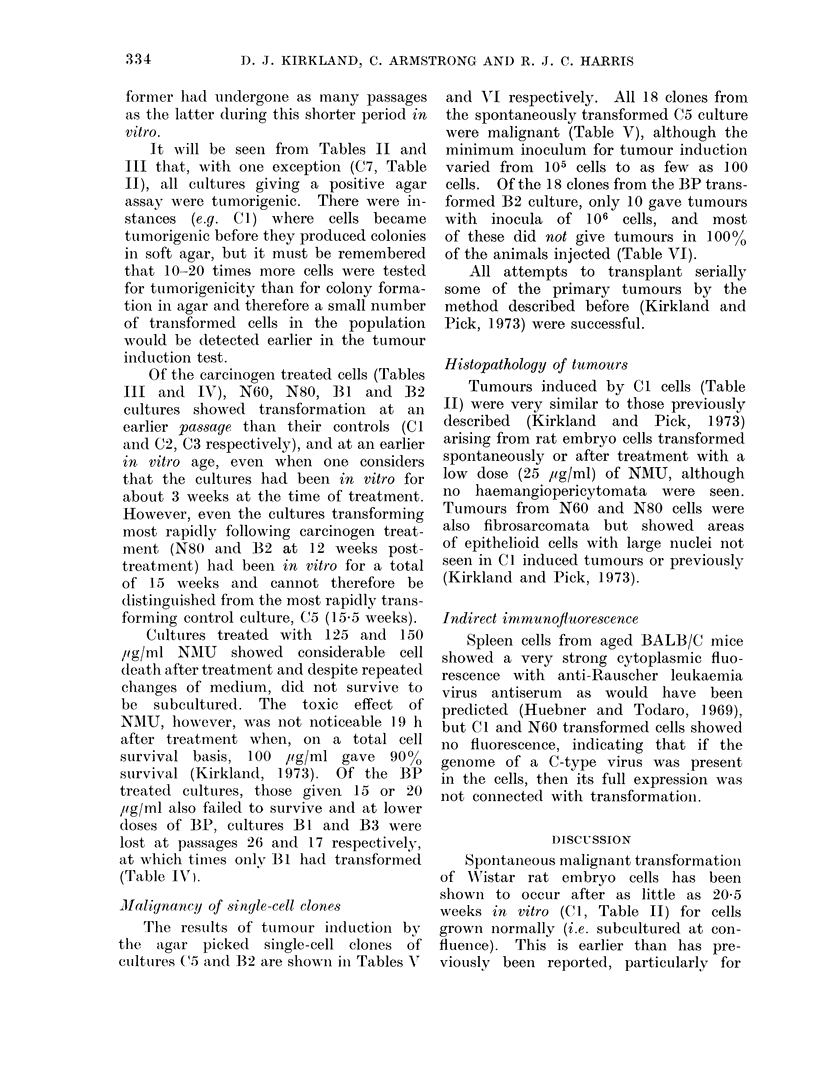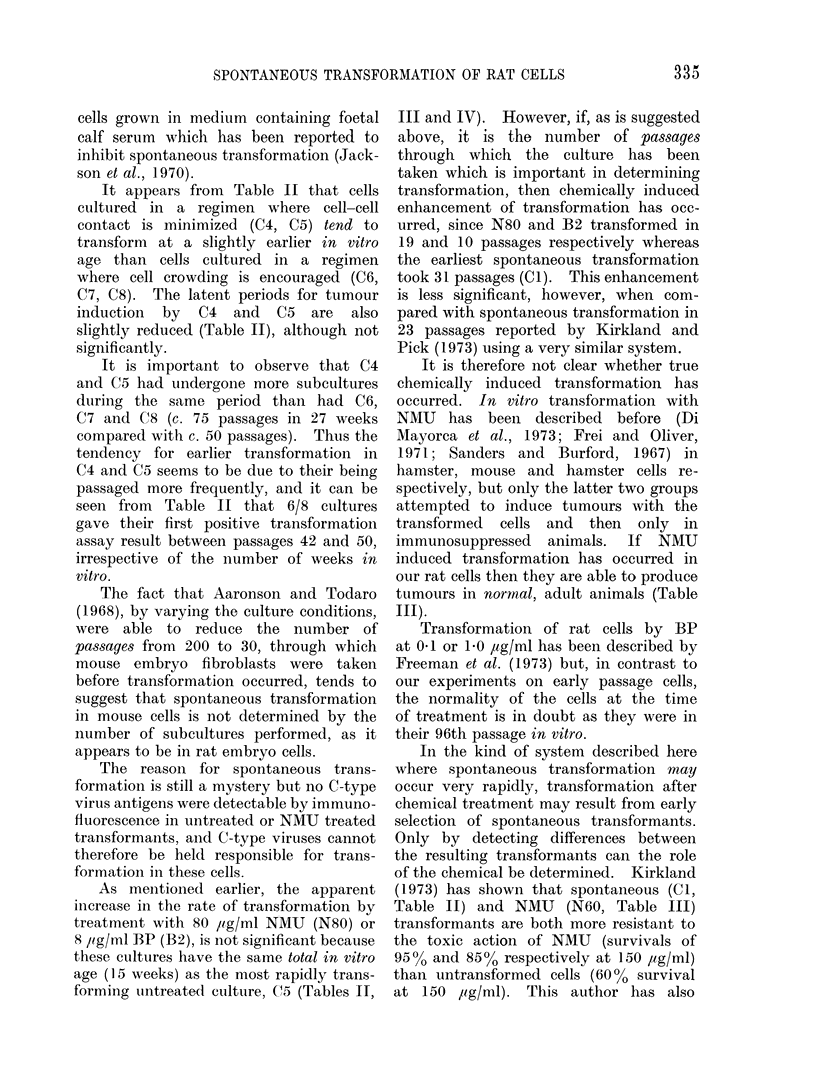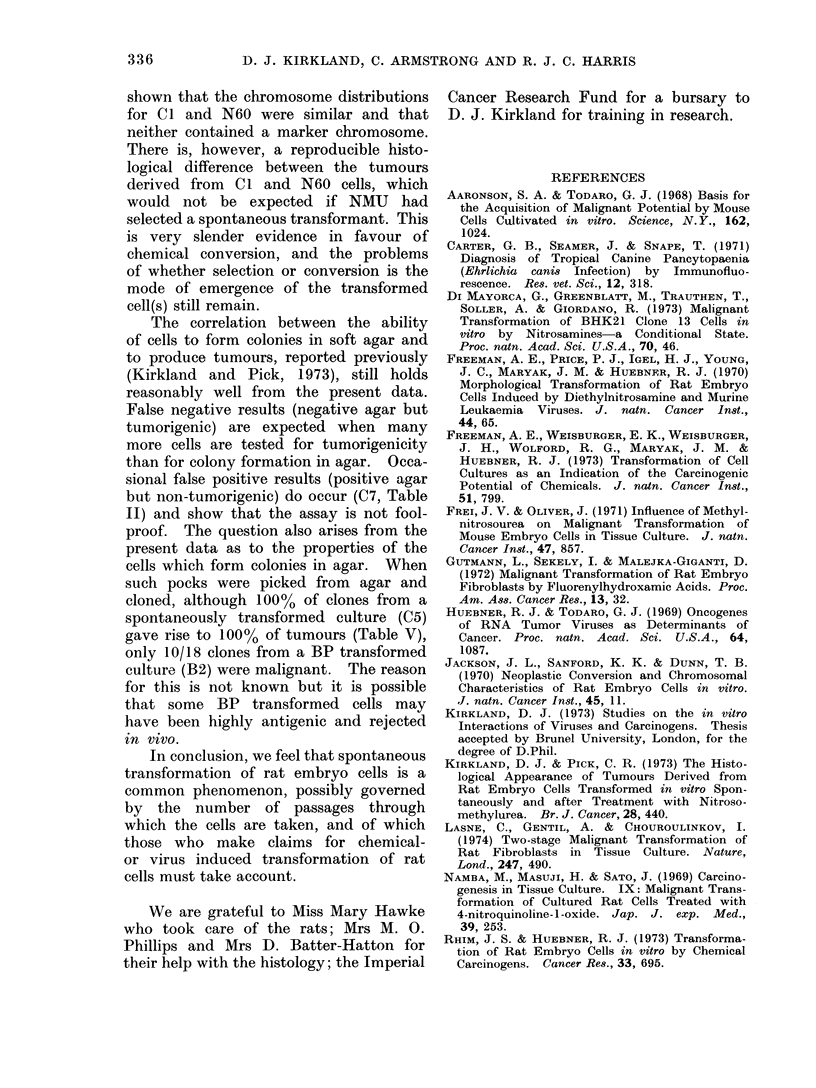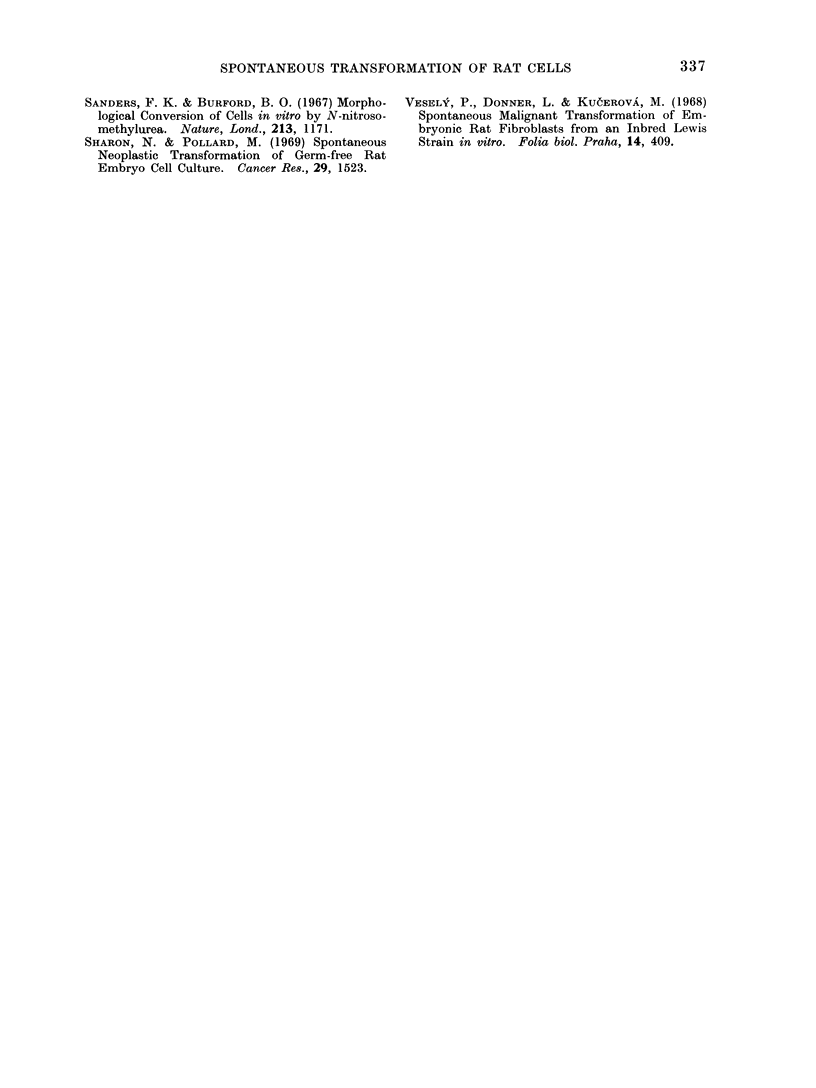# Spontaneous and chemically induced transformation of rat embryo cell cultures.

**DOI:** 10.1038/bjc.1975.67

**Published:** 1975-03

**Authors:** D. J. Kirkland, C. Armstrong, R. J. Harris

## Abstract

The transformation of Wistar rat embryo cells in vitro has been studied in passaged cultures using two criteria for transformation: (1) the ability of cells to form colonies in soft agar and (2) the ability of cells to form tumours in young syngeneic animals. In general there was good correlation between the two criteria. Spontaneous transformation was observed in all untreated cultures by 44 weeks although, by not allowing the cells to become confluent, the tendency was for cultures to transform earlier (i.e. 15-21 weeks). It was noticeable that despite untreated cultures having been in vitro for different lengths of time, most cultures transformed after a similar number of passages (42-50). Treatment of the embryo cells with the alkylating agent nitrosomethylurea (NMU) or benzo(alpha)pyrene (BP) sometimes resulted in transformation after a shorter period in vitro than the controls (minimum 12 weeks) although some treated cultures took longer. Transformed cells produced transplantable fibrosarcomata in syngeneic hosts and those arising from NMU transformed cells were histologically different from those arising from spontaneously transformed cells. The significant of spontaneous transformation in in vitro rat cell transformation systems is discussed.


*Br. J. Cancer* (1975) **31**, 329

### SPONTANEOUS AND CHEMICALLY INDUCED TRANSFORMATION OF RAT EMBRYO CELL CULTURES

D. J. KIRKLAND*, C. ARMSTRONG AND R. J. C. HARRIS†

*From the Imperial Cancer Research Fund, London NW7*

Received 19 September 1974. Accepted 28 October 1974

**Summary.—The transformation of Wistar rat embryo cells *in vitro* has been studied in passaged cultures using two criteria for transformation: (1) the ability of cells to form colonies in soft agar and (2) the ability of cells to form tumours in young syngeneic animals. In general there was good correlation between the two criteria. Spontaneous transformation was observed in all untreated cultures by 44 weeks although, by not allowing the cells to become confluent, the tendency was for cultures to transform earlier (*i.e.* 15–21 weeks). It was noticeable that despite untreated cultures having been *in vitro* for different lengths of time, most cultures transformed after a similar number of passages (42–50). Treatment of the embryo cells with the alkylating agent nitrosomethylurea (NMU) or benzo(α)pyrene (BP) sometimes resulted in transformation after a shorter period *in vitro* than the controls (minimum 12 weeks) although some treated cultures took longer. Transformed cells produced transplantable fibrosarcomata in syngeneic hosts and those arising from NMU transformed cells were histologically different from those arising from spontaneously transformed cells. The significance of spontaneous transformation in *in vitro* rat cell transformation systems is discussed.**

OUR INITIAL interest in chemical transformation of cells *in vitro* derived from attempts to obtain a more rapid estimate of the carcinogenicity of tobacco smoke condensates than could be obtained either by skin painting of tar or persuading mice to inhale a mixture of smoke and air, the former taking months and the latter years. Rat cells were preferable to mouse due to the prevalence of the ubiquitous leukaemia viruses in the latter and their tendency to transform spontaneously. Freeman *et al.* (1970) were unable to induce tumours in newborn rats with $10^6$ and $10^7$ rat embryo cells which had been grown for 58 subcultures, and Lasne, Gentil and Chouroulinkov (1974) were unable to get untreated rat fibroblasts to form either colonies in soft agar or tumours in newborn rats after 40 passages *in vitro* over 29 weeks.

Spontaneous transformation of *rat* cells has been reported by Veselý, Donner and Kučerová (1968) although in only 2 of 19 primary cultures; by Sharon and Pollard (1969) after 19 subcultures *in vitro*; by Jackson, Sanford and Dunn (1970) in 9–30 months, cells grown in the presence of horse serum transforming more readily than those grown in foetal calf serum; and by Kirkland and Pick (1973) after 23 passages. This paper shows that spontaneous transformation of rat cells is not a rare event, occurring in 8/8 untreated groups of cells by 44 weeks *in vitro*.

Aaronson and Todaro (1968) showed that the number of passages through which *mouse* embryo fibroblasts could be taken *in vitro* before transformation occurred could be reduced from 200 to 30 by altering the culture conditions from

* Present address: Institute of Cancer Research, Pollards Wood Research Station, Nightingales Lane, Chalfont St Giles, Buckinghamshire.

† Present address: Microbiological Research Establishment, Porton Down, Salisbury, Wiltshire.

minimal cell–cell contact with the cells always at low density to high cell–cell contact in thick monolayers. Our data suggest that the rate of spontaneous transformation of *rat* cells can also be enhanced by altering the culture conditions but, in contrast to the findings of Aaronson and Todaro (1968), from those allowing high cell–cell contact to those where the cells were never allowed to become confluent.

Chemical transformation of rat embryo cells in the absence of virus has been demonstrated for 4-nitroquinoline-1-oxide (Namba, Masuji and Sato, 1969), hydroxamic acids (Gutmann, Sekely and Malejka-Giganti, 1972) and polycyclic hydrocarbons (Rhim and Huebner, 1973). Kirkland and Pick (1973) showed that treatment of rat embryo cells with a low dose of nitrosomethylurea (NMU) did not enhance transformation above the spontaneous rate. This paper demonstrates that transformation of rat embryo cells *may sometimes* occur more rapidly following treatment with certain high doses of NMU or benzo($\alpha$)pyrene (BP), but that even these cases cannot be significantly distinguished from spontaneous transformations in untreated cultures. The problems of assessing chemically induced transformation in *in vitro* systems where spontaneous transformation occurs readily are discussed.

### MATERIALS AND METHODS

*Materials.*—Cells were grown in a complete medium (CM), except where stated, which consisted of Eagle's minimal essential medium supplemented with 10% autoclaved tryptose phosphate broth (Difco), 8% unheated calf serum (Fraburg, or MRE batches 821/71, 182/72, 573 IV/72 and 97E/73), 2% foetal bovine serum, 0·2% sodium bicarbonate (Analar), 100 i.u./ml penicillin, 100 $\mu$g/ml streptomycin and 2 $\mu$g/ml Fungizone (Squibb and Sons, New York). Control untreated and NMU treated cultures were maintained in 8 oz prescription bottles; BP treated cultures and untreated cultures tested for the effect of cell–cell contact on transformation were maintained in 10 cm and 6 cm plastic dishes (Esco AA) respectively. Apart from the cell–cell contact experiments, all cultures were split 1 : 5 by trypsinization when confluent (usually every 3–4 days).

The soft agar assay to detect transformed cells was, as described previously (Kirkland and Pick, 1973), carried out in 5 cm dishes (A/S Nunc, Denmark). The base layer comprised CM with 0·1% sodium bicarbonate and additional 0·5% Difco Noble agar, and the overlay was similar but contained 0·44% agar and 16% calf serum.

*Animals.*—Rats of the inbred Wistar strain were used. The colony, housed under minimal disease conditions at the Mill Hill laboratories of the Imperial Cancer Research Fund, was originally obtained from the Chester Beatty Research Institute.

*Cell culture.*—Cell cultures were derived from whole, 11-day rat embryos and were grown, in CM, at 37°C in a moist atmosphere of 5% $CO_2$ in air.

*Spontaneous transformation.*—One culture of mixed embryos (C1) and 2 cultures (C2, C3) from separate male embryos (sexed by examination under a dissecting microscope, and confirmed by karyotype analysis at an early passage *in vitro*) were propagated serially. In experiments to test the effect of the degree of cell–cell contact on transformation, a similar male embryo culture was split at the primary stage into 2 groups: C4 and C5 which were always seeded at $1·5 \times 10^5$ cells per 60 mm dish; C6, C7 and C8 which were always seeded at $6 \times 10^5$ cells/dish after subculture.

The rate of growth of the cells in these 2 groups was not always the same and therefore, although cell–cell contact was controlled to some degree by the different seeding densities mentioned above, it was sometimes necessary to reduce, and perhaps later increase, the rate of growth of the cells by altering the serum content of the medium. The changes in calf serum concentration that were necessary are shown in Table I.

### *Carcinogen treatment of rat embryo cultures*

*Nitrosomethylurea* (NMU).—Semi-confluent monolayers of cells in their third passage *in vitro* were treated, in duplicate bottles, with various concentrations (40, 50, 60, 70, 80, 100, 125 and 150 $\mu$g/ml) of NMU, freshly

TABLE I.—*Changes in Calf Serum Concentration of Medium*

| Passage no. | % concentration of calf serum: Culture C4 | Culture C5 | Cultures C6, C7, C8 |
| --- | --- | --- | --- |
| 1–3 | 4·5 | 4·5 | 4·5 |
| 4–16 | 2·0 | 2·0 | — |
| 4–18 | — | — | 2·5 |
| 17–21 | 4·5 | 4·5 | — |
| 19→ | — | — | 2·5+2% foetal |
| 22–42 | 8·0+2% foetal | — | — |
| 22–49 | — | 8·0+2% foetal | — |
| 43→ | 4·5 | — | — |
| 49→ | — | 4·5 | — |

prepared in phosphate buffered saline (PBS). After 2 h at 37°C the carcinogen was removed, the cells washed with fresh PBS, fed with CM and subcultured as described. Duplicate cultures that survived NMU treatment were combined giving cell lines N40, N50, N60, etc. The PBS treated control cell line, C1 (see above), was similarly derived.

*Benzo(α)pyrene* (BP).—BP, being insoluble in PBS, was first prepared as a colloidal suspension in aqueous gelatin by dissolving the BP in 1 ml warm acetone and adding dropwise to 25 ml of 0·5% gelatin which was gently stirred. This suspension was diluted 1/40 in CM to give the final solution.

The mode of treatment with BP was slightly different from that with NMU, in that cells in their second passage were plated at $5 \times 10^5$/100 mm Esco dish, and to each was added 10 ml of either control or BP containing medium thus:

| Cell culture | B1 | B2 | B3 | B4 | B5 | B6 |
| --- | --- | --- | --- | --- | --- | --- |
| Concentration of BP (μg/ml) | 4 | 8 | 5 | 10 | 15 | 20 |

For cultures B5, B6 the cells were first allowed to attach to the dish (4 h) and then 0·6 ml CM containing 24 μg DEAE dextran was added. The cells were incubated for 1 h, then washed with PBS and treated with BP as above.

After 5 days' incubation the control dishes were subcultured but BP treated cultures had very sparse monolayers with some fairly sick cells. However, the medium was removed and replaced with ordinary CM which was used throughout, with the glutamine present in the medium supplemented by an additional 300 mg/l.

*Assays for transformed cells*

(i) *Colony formation in soft agar.—All* cultures were tested every 3–4 passages for their ability to produce colonies in soft agar. Single-cell suspensions were made in 2 ml agar overlay medium at 40°C, which was then allowed to set on top of 6 ml of pre-set base medium. At each test $5 \times 10^4$, $10^4$ and $5 \times 10^3$ cells were plated in duplicate. Cultures were examined by microscopy to check if the overlay contained single cells or aggregates and those containing aggregates were discarded. Agar cultures were fed with 2 ml fresh overlay medium at 10 days and observed every 3–4 days for the appearance of colonies, which were counted at 21 days using either a hand lens or dissecting microscope. The counts were related to the number of cells plated to give an agar plating efficiency (APE).

(ii) *Induction of tumours.*—Young, adult, male Wistar rats were inoculated subcutaneously (s.c.) with suspensions of cells in CM (see Tables II, III, V and VI) from various passage levels. Sites of inoculation were palpated weekly for tumours. The animals were killed when s.c. growths reached 2 cm diameter, and examined at autopsy for gross metastases. Sections of some tumours were prepared for histological examination by C. R. Pick as described previously (Kirkland and Pick, 1973).

Rat embryo cells that produce colonies in agar have previously been found (Kirkland and Pick, 1973) to be tumorigenic and therefore, in these experiments, cultures were considered to be transformed if they gave a positive result in either of the 2 assays.

*Cloning of transformed cells from soft agar culture*

A fine scalpel was used to pick marked single colonies, which were then squashed physically on to the bottom of a 35 mm Falcon plastic dish; 2 ml CM were added and the dishes incubated for 1–3 weeks for the cells to attach and grow. Single colonies were then picked from a silicone greased ring and recultured.

Single-cell clones were thus established for C5 at 25 weeks (passage 73) and 27 weeks

TABLE II.—*Colony Formation in Agar and Tumour Induction by Untreated Rat Cells, from the Time of the First Positive Assay Result*¶

| Experiment | Passage no. | No. of weeks *in vitro* | Average APE (%)* | No. of tumours/ no. of animals inoculated† | Average latent period (weeks)‡ |
|---|---|---|---|---|---|
| C1 | 31 | 20·5 | 0 | 5/8 | 40 |
| | 35 | 22 | 0·01 | ND | — |
| | 45 | 27 | ND§ | 3/3 | 12 |
| C2 | 42 | 39·5 | ND | 2/2 | 10 |
| | 60 | 59 | ND | 3/3 | 10 |
| C3 | 36 | 44 | ND | 4/4 | 7 |
| | 58 | 57 | TNTC‖ | ND | — |
| Minimal cell–cell contact | | | | | |
| C4 | 55 | 21 | 0 | 2/2 | 10 |
| | 75 | 26·5 | ND | 3/3 | 6 |
| C5 | 45 | 15·5 | ND | 3/3 | 11 |
| | 63 | 21 | ND | 3/3 | 4 |
| High cell–cell contact | | | | | |
| C6 | 50 | 27 | 0·25 | ND | — |
| | 79 | 42·5 | ND | 3/3 | 18 |
| C7 | 44 | 24 | 0 | ND | — |
| | 68 | 36 | 4·0 | ND | — |
| | 74 | 40 | ND | 0/3 | — |
| C8 | 48 | 27 | 0 | ND | — |
| | 52 | 31·5 | ND | 3/3 | 20 |
| | 70 | 39 | 0·6 | ND | — |
| | 76 | 43 | ND | 3/3 | 14 |

* APE = agar plating efficiency = no. of colonies/no. of cells plated.
† Animals inoculated subcutaneously with $5 \times 10^5$–$10^6$ cells.
‡ Latent period = time taken from injection to tumour reaching approx. 2 cm diameter.
§ ND = Not done.
‖ TNTC = Too numerous to count.
¶ All previous tests were negative.

TABLE III.—*Colony Formation in Agar and Tumour Induction by NMU Treated Rat Cells, from the Time of the First Positive Assay Result*‡

| Experiment | Treatment (μg/ml NMU) | Passage no. | Weeks post-treatment | Average APE (%) | No. of tumours/ no. of animals inoculated* | Average latent period (weeks)* |
|---|---|---|---|---|---|---|
| N40 | 40 | 39 | 24 | 0·002 | ND* | — |
| N50 | 50 | 39 | 24 | 0·001 | ND | — |
| N60 | 60 | 23 | 14 | 0·003 | ND | — |
| | | 31 | 18 | 0·086 | 8/8 | 23 |
| | | 53 | 30 | ND | 3/3 | 9 |
| N70 | 70 | 31 | 20 | 0 | 0/8† | — |
| | | 39 | 24 | 0·002 | ND | — |
| N80 | 80 | 19 | 12 | 0·1 | ND | — |
| | | 31 | 18 | ND | 8/8 | 26 |
| | | 35 | 22 | 0·2 | ND | — |
| N100 | 100 | 39 | 24 | 0·002 | ND | — |

* See Table II.
† No tumours in 52 weeks.
‡ All previous tests were negative.

(passage 78), and B2 at 12 weeks (passage 10) and 17 weeks (passage 22) *in vitro*. Cells from each clone were inoculated s.c. into 6-week old male Wistar rats to determine (a) the minimum cell number required to produce tumours or (b) the latent period of tumour induction and tumour yield from an inoculum of $10^6$ cells.

*Indirect immunofluorescence.* — Transformed cells were examined for the presence of

TABLE IV.—*The First Detection of Transformed Rat Cells, Treated with Benzo(α)-pyrene, by Colony Formation in Agar*§

| Experiment | Treatment (μg/ml BP) | Passage no. | Weeks post-treatment | Average APE (%)* |
|---|---|---|---|---|
| B1 | 4† | 21 | 17 | 5·0 |
| | 0 | 17 | 17 | 0 |
| B2 | 8 | 10 | 12 | 0·1 |
| | 0 | 17 | 30 | 0 |
| B3 | 5‡ | 13 | 19·5 | 0 |
| | 0 | 43 | 34 | 0·1 |
| B4 | 10 | 23 | 24 | 0 |
| | 0 | 34 | 24 | TNTC* |

* See Table II.

† Lost at passage 26.

‡ Lost at passage 17.

§ All previous tests were negative.

TABLE V.—*Variation in the Minimum Numbers of Cells Required to Induce 100% Tumours within the Clones Derived from a Spontaneously Transformed Culture (C5)*

| Clone no. | Minimum tumour inducing dose (cells)* | Clone no. | Minimum tumour inducing dose (cells)* |
|---|---|---|---|
| 1 | $10^5$ | 10 | $10^4$ |
| 2 | $10^4$ | 11 | $10^2$ |
| 3 | $10^5$ | 12 | $10^4$ |
| 4 | $10^4$ | 13 | $10^3$ |
| 5 | $10^3$ | 14 | $10^3$ |
| 6 | $10^4$ | 15 | $10^4$ |
| 7 | $10^4$ | 16 | $10^2$ |
| 8 | $10^3$ | 17 | $10^2$ |
| 9 | $10^3$ | 18 | $10^3$ |

* The minimum number of cells giving rise to 100% tumours.

the Rauscher leukaemia virus gs antigen by indirect immunofluorescence, using rabbit anti-Rauscher leukaemia virus complement fixing antiserum (Virgo Reagents Ltd, Bethesda, Maryland, U.S.A.) as the first layer. The second layer was fluorescein labelled goat anti-rabbit globulin (Microbiological Associates, Bethesda, Maryland, U.S.A.), containing Evans blue stain to control nonspecific fluorescence as described by Carter, Seamer and Snape (1971). For a positive control, mashed spleens from aged BALB/C mice, which are reported to contain the gs complement fixing antigen (Huebner and Todaro, 1969), were treated similarly.

TABLE VI.—*Variation in the Malignancy of $10^6$ Injected Cells from Clones Derived from a Benzo[α]pyrene Transformed Culture (B2)*

| Clone no.* | No. of tumours/ no. of animals inoculated† | Latent period (weeks)‡ |
|---|---|---|
| 1 | 9/12 | 14–22 |
| 2 | 2/11 | 19–22 |
| 3 | 0/10§ | — |
| 4 | 0/7§ | — |
| 5 | 0/6§ | — |
| 6 | 1/13 | 21 |
| 7 | 2/2 | 2 |
| 8 | 8/8 | 11–21 |
| 9 | 10/10 | 10–16 |
| 10 | 4/7 | 22–29 |
| 11 | 0/10§ | — |
| 12 | 1/8 | 20 |
| 13 | 2/10 | 15–20 |
| 14 | 1/10 | 15 |
| 15 | 0/8‖ | — |
| 16 | 0/10¶ | — |
| 17 | 0/9¶ | — |
| 18 | 0/8¶ | — |

* Clones 1–10 established 12 weeks post treatment; clones 11–18 established 22 weeks post treatment.
† All animals inoculated subcutaneously with $10^6$ cells.
‡ Latent period = time taken from injection for tumour to reach approx. 2 cm diameter.
§ No tumours after 21 weeks.
‖ No tumours after 20 weeks.
¶ No tumours after 19 weeks.

### RESULTS

### *Assays for transformed cells*

The results of colony formation in agar and tumour induction, from the passage at which a positive result was first obtained (all assays at earlier passages were negative), are shown in Tables II, III and IV.

Some untreated cultures (Table II) transformed after only 16–21 weeks *in vitro* (C1, C4 and C5) whereas others took much longer (C2, C3, C6, C7 and C8) but all had transformed by 44 weeks *in vitro*, either by showing colony formation in agar or induction of tumours *in vivo*. Cultures in which cell–cell contact was minimal (C4, C5), showed a definite trend towards earlier transformation than those cultures where cell–cell contact was high (C6, C7 and C8), although the former had undergone as many passages as the latter during this shorter period *in vitro*.

It will be seen from Tables II and III that, with one exception (C7, Table II), all cultures giving a positive agar assay were tumorigenic. There were instances (*e.g.* C1) where cells became tumorigenic before they produced colonies in soft agar, but it must be remembered that 10–20 times more cells were tested for tumorigenicity than for colony formation in agar and therefore a small number of transformed cells in the population would be detected earlier in the tumour induction test.

Of the carcinogen treated cells (Tables III and IV), N60, N80, B1 and B2 cultures showed transformation at an earlier *passage* than their controls (C1 and C2, C3 respectively), and at an earlier *in vitro* age, even when one considers that the cultures had been *in vitro* for about 3 weeks at the time of treatment. However, even the cultures transforming most rapidly following carcinogen treatment (N80 and B2 at 12 weeks post-treatment) had been *in vitro* for a total of 15 weeks and cannot therefore be distinguished from the most rapidly transforming control culture, C5 (15·5 weeks).

Cultures treated with 125 and 150 μg/ml NMU showed considerable cell death after treatment and despite repeated changes of medium, did not survive to be subcultured. The toxic effect of NMU, however, was not noticeable 19 h after treatment when, on a total cell survival basis, 100 μg/ml gave 90% survival (Kirkland, 1973). Of the BP treated cultures, those given 15 or 20 μg/ml also failed to survive and at lower doses of BP, cultures B1 and B3 were lost at passages 26 and 17 respectively, at which times only B1 had transformed (Table IV).

*Malignancy of single-cell clones*

The results of tumour induction by the agar picked single-cell clones of cultures C5 and B2 are shown in Tables V and VI respectively. All 18 clones from the spontaneously transformed C5 culture were malignant (Table V), although the minimum inoculum for tumour induction varied from $10^5$ cells to as few as 100 cells. Of the 18 clones from the BP transformed B2 culture, only 10 gave tumours with inocula of $10^6$ cells, and most of these did *not* give tumours in 100% of the animals injected (Table VI).

All attempts to transplant serially some of the primary tumours by the method described before (Kirkland and Pick, 1973) were successful.

*Histopathology of tumours*

Tumours induced by C1 cells (Table II) were very similar to those previously described (Kirkland and Pick, 1973) arising from rat embryo cells transformed spontaneously or after treatment with a low dose (25 μg/ml) of NMU, although no haemangiopericytomata were seen. Tumours from N60 and N80 cells were also fibrosarcomata but showed areas of epithelioid cells with large nuclei not seen in C1 induced tumours or previously (Kirkland and Pick, 1973).

*Indirect immunofluorescence*

Spleen cells from aged BALB/C mice showed a very strong cytoplasmic fluorescence with anti-Rauscher leukaemia virus antiserum as would have been predicted (Huebner and Todaro, 1969), but C1 and N60 transformed cells showed no fluorescence, indicating that if the genome of a C-type virus was present in the cells, then its full expression was not connected with transformation.

### DISCUSSION

Spontaneous malignant transformation of Wistar rat embryo cells has been shown to occur after as little as 20·5 weeks *in vitro* (C1, Table II) for cells grown normally (*i.e.* subcultured at confluence). This is earlier than has previously been reported, particularly for cells grown in medium containing foetal calf serum which has been reported to inhibit spontaneous transformation (Jackson *et al.*, 1970).

It appears from Table II that cells cultured in a regimen where cell–cell contact is minimized (C4, C5) *tend* to transform at a slightly earlier *in vitro* age than cells cultured in a regimen where cell crowding is encouraged (C6, C7, C8). The latent periods for tumour induction by C4 and C5 are also slightly reduced (Table II), although not significantly.

It is important to observe that C4 and C5 had undergone more subcultures during the same period than had C6, C7 and C8 (*c.* 75 passages in 27 weeks compared with *c.* 50 passages). Thus the tendency for earlier transformation in C4 and C5 seems to be due to their being passaged more frequently, and it can be seen from Table II that 6/8 cultures gave their first positive transformation assay result between passages 42 and 50, irrespective of the number of weeks *in vitro.*

The fact that Aaronson and Todaro (1968), by varying the culture conditions, were able to reduce the number of *passages* from 200 to 30, through which mouse embryo fibroblasts were taken before transformation occurred, tends to suggest that spontaneous transformation in mouse cells is not determined by the number of subcultures performed, as it appears to be in rat embryo cells.

The reason for spontaneous transformation is still a mystery but no C-type virus antigens were detectable by immunofluorescence in untreated or NMU treated transformants, and C-type viruses cannot therefore be held responsible for transformation in these cells.

As mentioned earlier, the apparent increase in the rate of transformation by treatment with 80 μg/ml NMU (N80) or 8 μg/ml BP (B2), is not significant because these cultures have the same *total in vitro* age (15 weeks) as the most rapidly transforming untreated culture, C5 (Tables II, III and IV). However, if, as is suggested above, it is the number of *passages* through which the culture has been taken which is important in determining transformation, then chemically induced enhancement of transformation has occurred, since N80 and B2 transformed in 19 and 10 passages respectively whereas the earliest spontaneous transformation took 31 passages (C1). This enhancement is less significant, however, when compared with spontaneous transformation in 23 passages reported by Kirkland and Pick (1973) using a very similar system.

It is therefore not clear whether true chemically induced transformation has occurred. *In vitro* transformation with NMU has been described before (Di Mayorca *et al.*, 1973; Frei and Oliver, 1971; Sanders and Burford, 1967) in hamster, mouse and hamster cells respectively, but only the latter two groups attempted to induce tumours with the transformed cells and then only in immunosuppressed animals. If NMU induced transformation has occurred in our rat cells then they are able to produce tumours in *normal*, adult animals (Table III).

Transformation of rat cells by BP at 0·1 or 1·0 μg/ml has been described by Freeman *et al.* (1973) but, in contrast to our experiments on early passage cells, the normality of the cells at the time of treatment is in doubt as they were in their 96th passage *in vitro.*

In the kind of system described here where spontaneous transformation *may* occur very rapidly, transformation after chemical treatment may result from early selection of spontaneous transformants. Only by detecting differences between the resulting transformants can the role of the chemical be determined. Kirkland (1973) has shown that spontaneous (C1, Table II) and NMU (N60, Table III) transformants are both more resistant to the toxic action of NMU (survivals of 95% and 85% respectively at 150 μg/ml) than untransformed cells (60% survival at 150 μg/ml). This author has also shown that the chromosome distributions for C1 and N60 were similar and that neither contained a marker chromosome. There is, however, a reproducible histological difference between the tumours derived from C1 and N60 cells, which would not be expected if NMU had selected a spontaneous transformant. This is very slender evidence in favour of chemical conversion, and the problems of whether selection or conversion is the mode of emergence of the transformed cell(s) still remain.

The correlation between the ability of cells to form colonies in soft agar and to produce tumours, reported previously (Kirkland and Pick, 1973), still holds reasonably well from the present data. False negative results (negative agar but tumorigenic) are expected when many more cells are tested for tumorigenicity than for colony formation in agar. Occasional false positive results (positive agar but non-tumorigenic) do occur (C7, Table II) and show that the assay is not foolproof. The question also arises from the present data as to the properties of the cells which form colonies in agar. When such pocks were picked from agar and cloned, although 100% of clones from a spontaneously transformed culture (C5) gave rise to 100% of tumours (Table V), only 10/18 clones from a BP transformed culture (B2) were malignant. The reason for this is not known but it is possible that some BP transformed cells may have been highly antigenic and rejected *in vivo*.

In conclusion, we feel that spontaneous transformation of rat embryo cells is a common phenomenon, possibly governed by the number of passages through which the cells are taken, and of which those who make claims for chemical- or virus induced transformation of rat cells must take account.

We are grateful to Miss Mary Hawke who took care of the rats; Mrs M. O. Phillips and Mrs D. Batter-Hatton for their help with the histology; the Imperial Cancer Research Fund for a bursary to D. J. Kirkland for training in research.

### REFERENCES

AARONSON, S. A. & TODARO, G. J. (1968) Basis for the Acquisition of Malignant Potential by Mouse Cells Cultivated *in vitro*. *Science, N.Y.*, **162**, 1024.

CARTER, G. B., SEAMER, J. & SNAPE, T. (1971) Diagnosis of Tropical Canine Pancytopaenia (*Ehrlichia canis* Infection) by Immunofluorescence. *Res. vet. Sci.*, **12**, 318.

DI MAYORCA, G., GREENBLATT, M., TRAUTHEN, T., SOLLER, A. & GIORDANO, R. (1973) Malignant Transformation of BHK21 Clone 13 Cells *in vitro* by Nitrosamines—a Conditional State. *Proc. natn. Acad. Sci. U.S.A.*, **70**, 46.

FREEMAN, A. E., PRICE, P. J., IGEL, H. J., YOUNG, J. C., MARYAK, J. M. & HUEBNER, R. J. (1970) Morphological Transformation of Rat Embryo Cells Induced by Diethylnitrosamine and Murine Leukaemia Viruses. *J. natn. Cancer Inst.*, **44**, 65.

FREEMAN, A. E., WEISBURGER, E. K., WEISBURGER, J. H., WOLFORD, R. G., MARYAK, J. M. & HUEBNER, R. J. (1973) Transformation of Cell Cultures as an Indication of the Carcinogenic Potential of Chemicals. *J. natn. Cancer Inst.*, **51**, 799.

FREI, J. V. & OLIVER, J. (1971) Influence of Methylnitrosourea on Malignant Transformation of Mouse Embryo Cells in Tissue Culture. *J. natn. Cancer Inst.*, **47**, 857.

GUTMANN, L., SEKELY, I. & MALEJKA-GIGANTI, D. (1972) Malignant Transformation of Rat Embryo Fibroblasts by Fluorenylhydroxamic Acids. *Proc. Am. Ass. Cancer Res.*, **13**, 32.

HUEBNER, R. J. & TODARO, G. J. (1969) Oncogenes of RNA Tumor Viruses as Determinants of Cancer. *Proc. natn. Acad. Sci. U.S.A.*, **64**, 1087.

JACKSON, J. L., SANFORD, K. K. & DUNN, T. B. (1970) Neoplastic Conversion and Chromosomal Characteristics of Rat Embryo Cells *in vitro*. *J. natn. Cancer Inst.*, **45**, 11.

KIRKLAND, D. J. (1973) Studies on the *in vitro* Interactions of Viruses and Carcinogens. Thesis accepted by Brunel University, London, for the degree of D.Phil.

KIRKLAND, D. J. & PICK, C. R. (1973) The Histological Appearance of Tumours Derived from Rat Embryo Cells Transformed *in vitro* Spontaneously and after Treatment with Nitrosomethylurea. *Br. J. Cancer*, **28**, 440.

LASNE, C., GENTIL, A. & CHOUROULINKOV, I. (1974) Two-stage Malignant Transformation of Rat Fibroblasts in Tissue Culture. *Nature, Lond.*, **247**, 490.

NAMBA, M., MASUJI, H. & SATO, J. (1969) Carcinogenesis in Tissue Culture. IX: Malignant Transformation of Cultured Rat Cells Treated with 4-nitroquinoline-1-oxide. *Jap. J. exp. Med.*, **39**, 253.

RHIM, J. S. & HUEBNER, R. J. (1973) Transformation of Rat Embryo Cells *in vitro* by Chemical Carcinogens. *Cancer Res.*, **33**, 695.

SANDERS, F. K. & BURFORD, B. O. (1967) Morphological Conversion of Cells *in vitro* by *N*-nitrosomethylurea. *Nature, Lond.*, **213**, 1171.
SHARON, N. & POLLARD, M. (1969) Spontaneous Neoplastic Transformation of Germ-free Rat Embryo Cell Culture. *Cancer Res.*, **29**, 1523.
VESELÝ, P., DONNER, L. & KUČEROVÁ, M. (1968) Spontaneous Malignant Transformation of Embryonic Rat Fibroblasts from an Inbred Lewis Strain *in vitro*. *Folia biol. Praha*, **14**, 409.